# Augmented Repair of an Acute Midsubstance Patellar Tendon Rupture Using Suture Anchors, InternalBrace™, and Achilles Tendon Allograft: A Case Report

**DOI:** 10.7759/cureus.106003

**Published:** 2026-03-27

**Authors:** Iturbide A Ponce de Leon Sandoval, Nolasco Alejandro Robles Hernández, Jacobo Kerbel Sutton, Emilio Remolina Sánchez Rucobo, José Castillo de la Peña

**Affiliations:** 1 Orthopedics, Orthopedics and Traumatology Center, American British Cowdray (ABC) Medical Center, Mexico City, MEX; 2 Sports Medicine, American British Cowdray (ABC) Medical Center, Mexico City, MEX

**Keywords:** achilles tendon allograft, augmentation, extensor mechanism, internal brace, knee rehabilitation, midsubstance rupture, patellar tendon rupture, suture anchor, tendon repair

## Abstract

Patellar tendon (PT) ruptures are rare but disabling injuries that result in loss of active knee extension and significant functional impairment. We present the case of a 56-year-old male who sustained a complete midsubstance PT rupture after a direct blow to the knee. MRI confirmed the injury, and surgical management included end-to-end repair with suture anchors, augmentation with an *Internal*Brace™ (Arthrex Inc, Naples, USA), circumferential Achilles tendon allograft reinforcement, and repair of the medial and lateral patellofemoral capsule. Postoperative imaging confirmed restoration of patellar height, and early rehabilitation was initiated using a hinged knee brace. Augmented repair provided robust biomechanical stability, preserved patellar height, and allowed progressive early mobilization. Clinical outcomes for acute midsubstance PT ruptures demonstrate high rates of functional recovery and return to daily activities, sport, and work. Acute midsubstance PT ruptures can thus be effectively managed with combined repair and augmentation strategies, achieving excellent functional outcomes while enabling early rehabilitation.

## Introduction

Patellar tendon (PT) rupture is a rare but highly disabling injury, resulting in loss of active knee extension and significant functional impairment [1-3]. The injury typically occurs in young to middle-aged adults, with peak incidence in the third and fourth decades of life [3-4]. While less common in older patients, systemic conditions such as diabetes, chronic renal failure, or corticosteroid use may predispose to tendon rupture [5]. According to Tandogan et al., early surgical repair within the first few weeks following injury is recommended, as delayed treatment results in inferior outcomes and may require more complex reconstructions [6].

The most frequent mechanism of PT rupture is a forceful eccentric contraction of the quadriceps with the knee in flexion, often occurring during sports activities or sudden deceleration [1-2,5]. In athletes, ruptures are typically non-contact, whereas direct trauma may occasionally contribute to injury and can be associated with concomitant quadriceps tendon tears [1]. Clinically, patients report acute pain, a popping sensation, inability to actively extend the knee, and a palpable infrapatellar gap [2]. Unilateral patella alta and an Insall-Salvati index over 1.2 are suggestive of complete tears [6].

Most PT ruptures occur at the inferior pole of the patella, followed by mid-substance and tibial tubercle levels [3,7]. Ruptures near the patellar insertion are commonly amenable to primary repair, whereas mid-substance tears may require more complex suture techniques or augmentation [1-3]. Three main injury mechanisms have been described: indirect low-energy trauma, typically after eccentric contraction of the quadriceps; indirect high-energy trauma, often associated with multi-ligamentous knee injury; and direct trauma, including penetrating injuries or iatrogenic damage [6].

Primary repair is considered the standard for acute PT ruptures, ideally performed within two weeks of injury, using either transosseous sutures or suture anchors to restore the anatomic attachment of the tendon [2-3,7]. A comparative study has reported lower re-rupture rates with suture anchor repair compared to transosseous techniques (0% vs. 7.5%, respectively) [7], although these findings may not be generalizable across all clinical scenarios. Biomechanical studies demonstrate that suture anchors provide less gap formation under cyclic loading while maintaining similar ultimate load to failure compared with transosseous techniques, facilitating early mobilization [5,8].

In cases with poor tissue quality, tendinosis, or tendon retraction, augmentation using autografts (e.g., semitendinosus or gracilis) or cerclage wiring/polydioxanone suture (PDS) cords increases construct strength, reduces elongation under cyclic loading, and lowers re-rupture risk [2,4,8]. Novel techniques such as knotless suture anchor *Internal*Brace™ (SAIB; *Internal*Brace™ by Arthrex Inc, Naples, USA) augmentation provide biomechanical equivalence to traditional cerclage wire, improve yield and ultimate load, and allow accelerated postoperative rehabilitation [4]. Earlier reports by Larson and Simonian demonstrated that semitendinosus tendon augmentation in acute patellar tendon repair could provide sufficient protection of the repair to enable immediate mobilization without compromising tendon healing [9]. Their findings supported the concept of biologically and mechanically augmented constructs that facilitate early rehabilitation and functional recovery. Chronic or neglected tears may require augmentation with autograft or allograft tendons, with hamstrings or fresh-frozen Achilles tendon grafts being most commonly used [6].

Postoperative rehabilitation emphasizes protected early range of motion, progressive weight bearing, and quadriceps strengthening. Clinical studies demonstrate that primary repair, especially when augmented, reliably restores patellar height and knee function, with low rates of extensor lag and re-rupture [2-3]. Although return to play and work after PT repair is generally high, many patients do not achieve their pre-injury level of activity [10]. Overall, acute repair of patellar tendon ruptures using modern augmentation techniques offers excellent functional outcomes, low complication rates, and the ability to initiate early rehabilitation, minimizing long-term disability.

In chronic or complex cases, such as those involving delayed treatment, degenerative rupture, or poor tissue quality, direct repair may not be feasible, and reconstruction of the extensor mechanism becomes necessary to restore function [11]. Existing evidence suggests that biologically and mechanically augmented constructs may contribute to improved structural integrity and functional outcomes, particularly in complex extensor mechanism injuries or in the setting of poor tissue quality [11].

## Case presentation

A 56-year-old male presented to the emergency department after sustaining a ground-level fall resulting in a direct impact to the right knee while it was flexed. The patient immediately experienced acute pain, swelling, and inability to actively extend the knee. He was unable to bear weight or ambulate following the injury.

On physical examination, the right knee demonstrated diffuse swelling and joint effusion. Palpation revealed a palpable defect along the course of the patellar tendon. Active knee extension was absent, consistent with disruption of the extensor mechanism. Passive range of motion was limited to 20° of flexion and 0° of extension due to pain.

**Figure 1:**
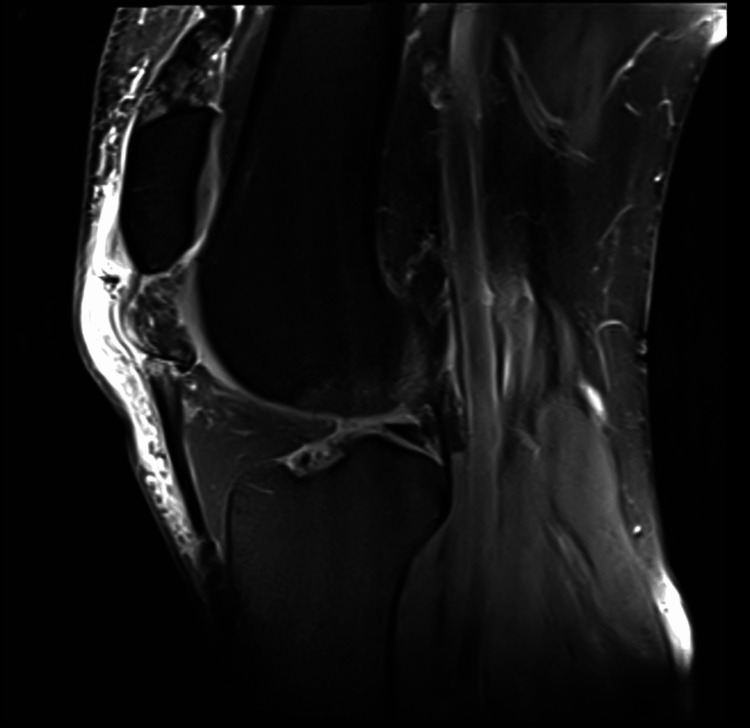
Sagittal MRI demonstrating midsubstance patellar tendon rupture. Sagittal T2-weighted fat-suppressed magnetic resonance imaging (MRI) of the right knee demonstrating a complete midsubstance rupture of the patellar tendon, with proximal displacement of the patella and surrounding soft tissue edema.

**Figure 2:**
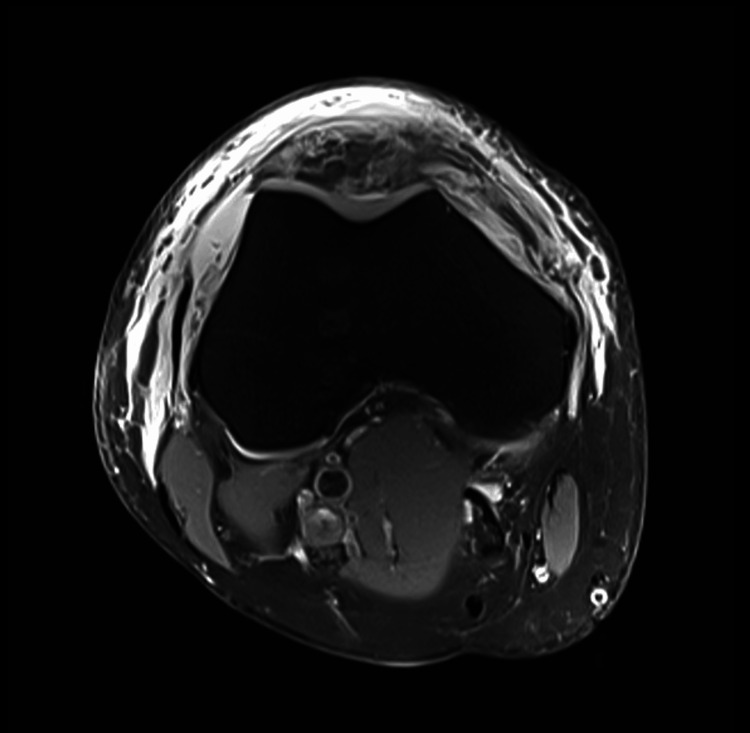
Axial MRI demonstrating associated extensor mechanism injury. Axial T2-weighted fat-suppressed MRI demonstrating associated soft tissue injury with edema and disruption of the lateral patellofemoral retinaculum and surrounding extensor mechanism structures.

Plain radiographs of the right knee demonstrated patella alta without evidence of acute fracture. Magnetic resonance imaging (MRI) confirmed a complete midsubstance rupture of the patellar tendon with proximal displacement of the patella (Figure 1). Additional findings included complete rupture of the lateral patellofemoral retinaculum, intrasubstance tearing of the medial retinaculum, and associated edema with partial tearing of the vastus medialis and vastus lateralis musculature (Figure 2).

Based on these findings, a diagnosis of complete rupture of the knee extensor mechanism secondary to midsubstance patellar tendon rupture was established, and surgical repair was indicated.

**Figure 3:**
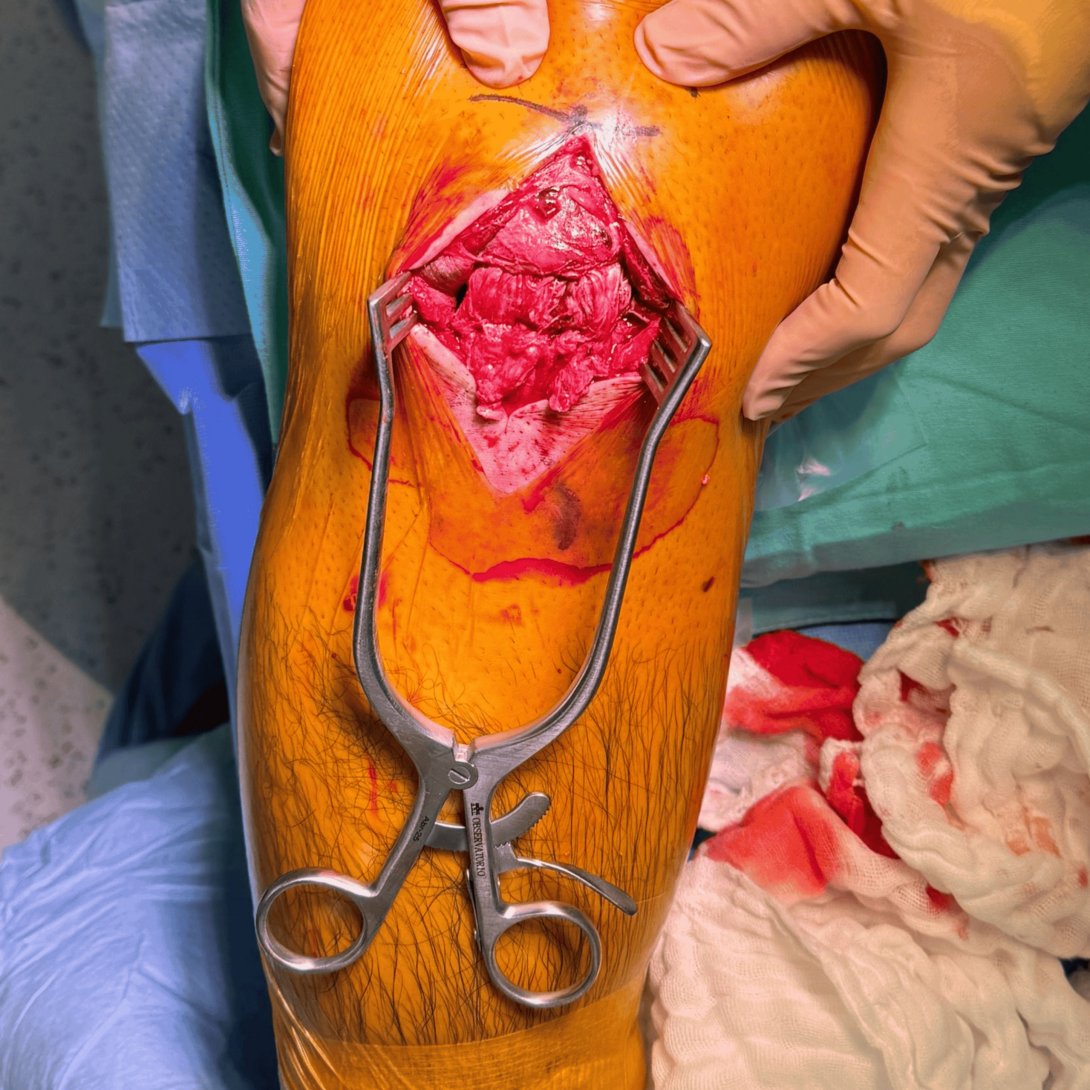
Intraoperative view of midsubstance patellar tendon rupture. Intraoperative photograph demonstrating a complete midsubstance rupture of the patellar tendon with poor tissue quality and gapping at the rupture site prior to repair.

Surgery was performed with the patient in the supine position on the operating table under sedation and regional anesthesia, with antibiotic prophylaxis administered preoperatively. A midline longitudinal incision was made over the anterior knee to expose the extensor mechanism. Intraoperative evaluation confirmed a complete midsubstance rupture of the patellar tendon with poor tissue quality and gapping at the rupture site (Figure 3). Nonviable tissue at the tendon edges was carefully debrided.

**Figure 4:**
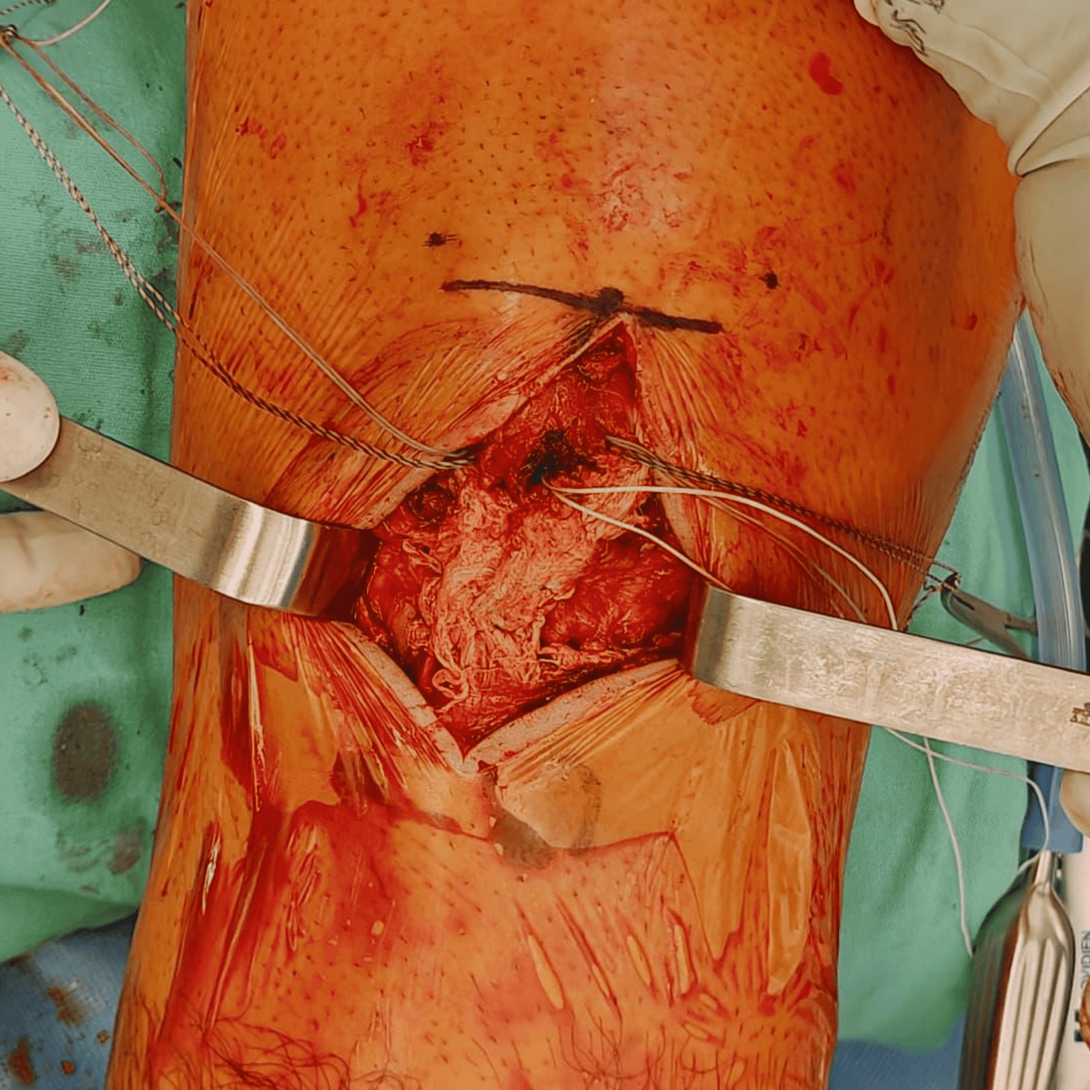
Suture anchor placement at the distal pole of the patella. Intraoperative image demonstrating placement of two suture anchors (Trushot loaded with Y-Knot sutures) at the distal pole of the patella in a parallel configuration to facilitate end-to-end patellar tendon repair.

The distal pole of the patella was prepared for anchor placement, and two all-suture anchors (TruShot®, loaded with Y-Knot® sutures; Conmed Corporation, Utica, USA) were inserted in a parallel configuration (Figure 4). An end-to-end repair of the patellar tendon was then performed using a Krackow locking suture technique on both the medial and lateral tendon segments. Intraoperative fluoroscopy was used to confirm restoration of patellar height using the contralateral knee as a reference.

**Figure 5:**
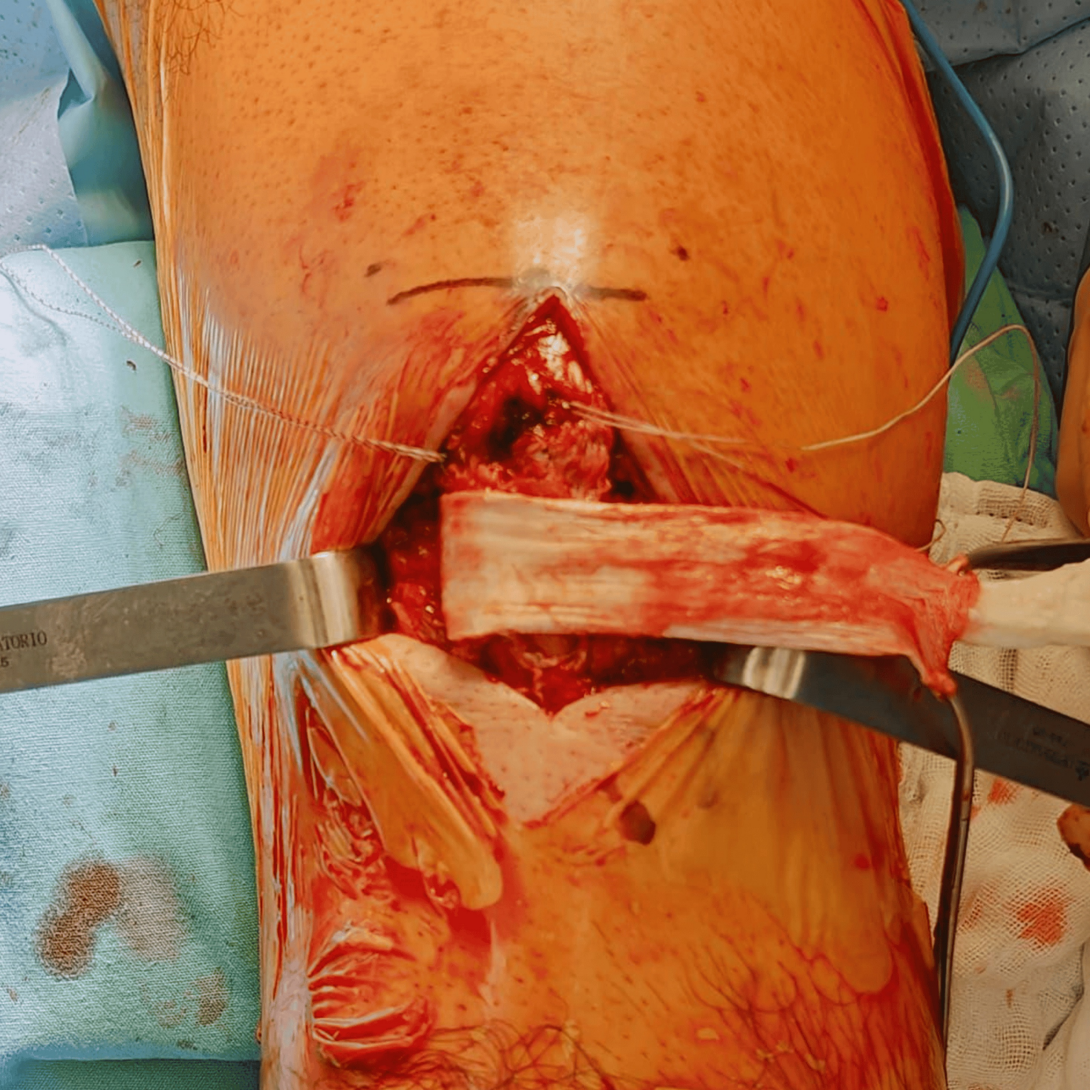
Circumferential Achilles tendon allograft augmentation. Intraoperative image demonstrating circumferential placement of an Achilles tendon allograft around the repaired patellar tendon to provide biological augmentation of the repair construct.

**Figure 6:**
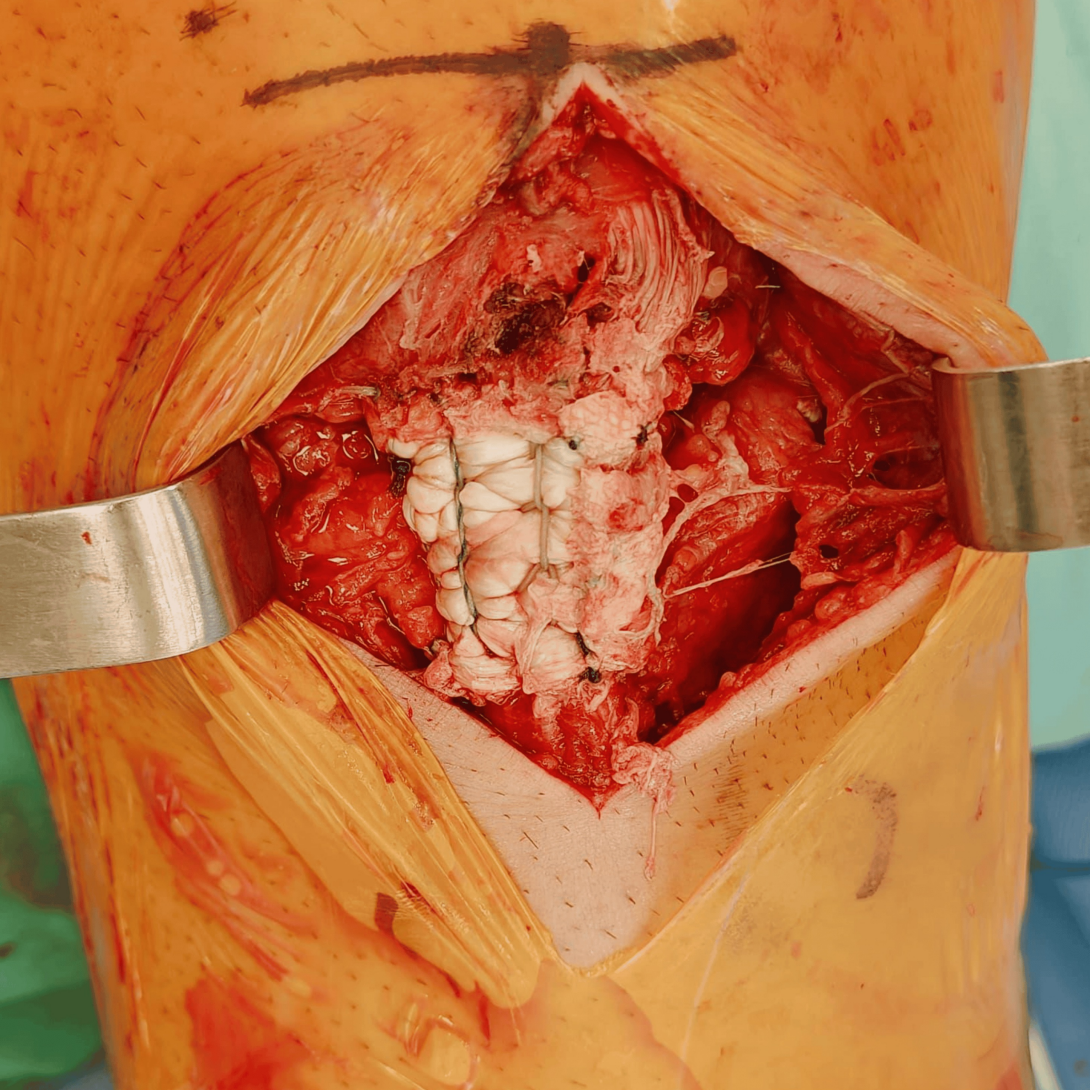
Integration of the allograft with the native tendon repair. Intraoperative image demonstrating integration of the Achilles tendon allograft with the native patellar tendon repair using residual sutures from the previously placed suture anchors.

An *Internal*Brace™ construct was then placed beneath the repair using two SwiveLock anchors (Arthrex Inc, Naples, USA), with controlled tension applied to support the repair while avoiding overtensioning of the construct and patellofemoral overconstraint. Due to the observed poor tissue quality, biological augmentation was performed using a BioCleanse® Achilles tendon allograft (RTI Surgical, Alachua, Florida). The graft was wrapped circumferentially around the repaired tendon (Figure 5) and secured using nonabsorbable sutures. Residual sutures from the anchors were used to secure the allograft to the native tendon repair, reinforcing the construct and facilitating integration between tissues (Figure 6).

**Figure 7:**
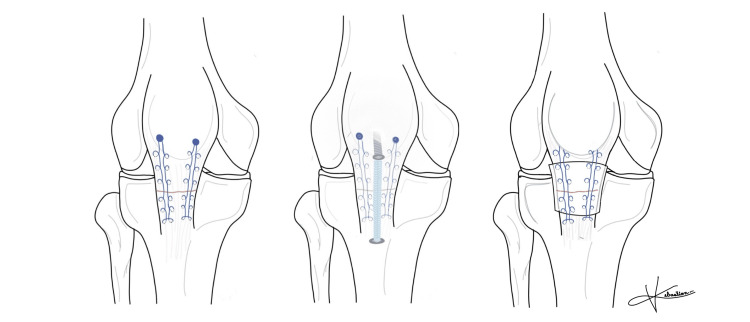
Schematic representation of the final repair construct. Schematic illustration of the final repair construct demonstrating distal patellar suture anchor fixation, *Internal*Brace™ (Arthrex Inc, Naples, USA) augmentation, and circumferential Achilles tendon allograft reinforcement. Illustration manually hand-drawn by Alonso Sebastián Villegas Treviño.

Finally, the medial and lateral patellofemoral retinacular structures were repaired to restore soft tissue stability. The completed construct demonstrated stable tendon apposition and restoration of extensor mechanism continuity. Figure 7 illustrates a schematic representation of the final repair construct, including the suture anchors, *Internal*Brace™ augmentation, and circumferential Achilles tendon allograft reinforcement.

**Figure 8:**
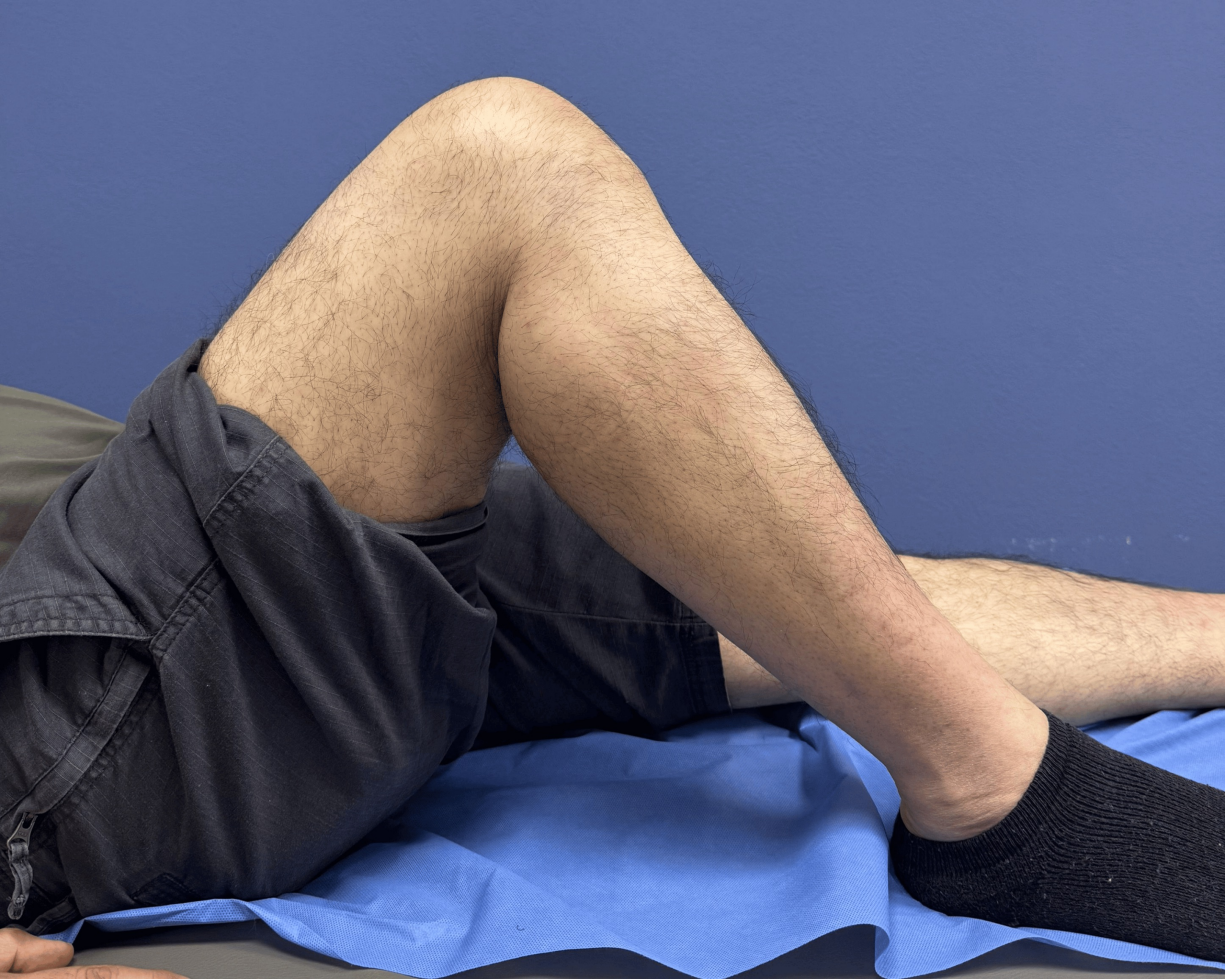
Postoperative knee flexion at seven weeks. Clinical photograph obtained seven weeks postoperatively demonstrating satisfactory knee flexion following augmented patellar tendon repair.

**Figure 9:**
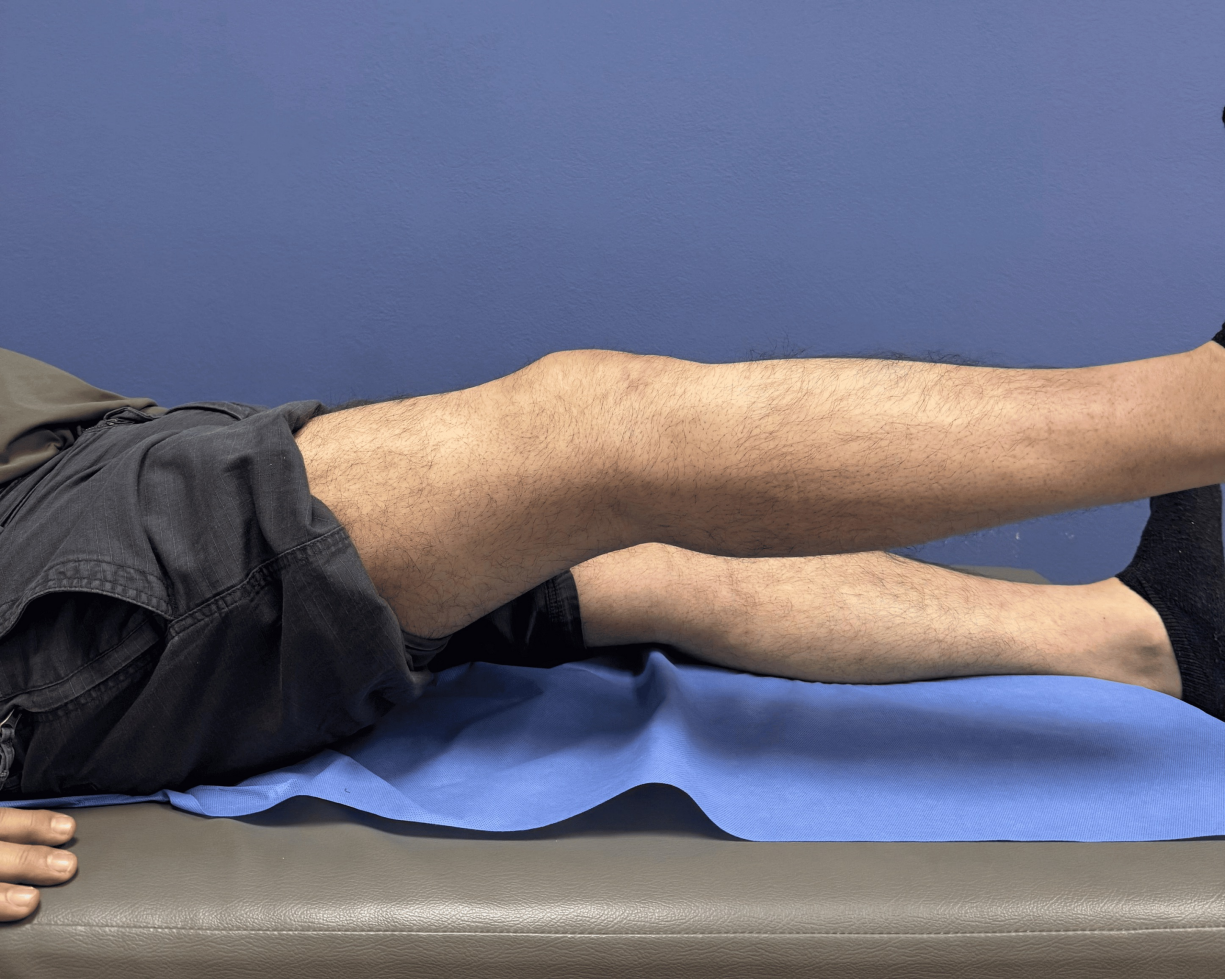
Postoperative knee extension at seven weeks. Clinical photograph obtained seven weeks postoperatively demonstrating near-full knee extension with minimal residual extension lag.

Postoperatively, the knee was immobilized in full extension using a hinged knee brace. Progressive rehabilitation was initiated with a protected range of motion. At three weeks, knee flexion had progressed to 60° with the brace in place. At seven weeks, the patient achieved 95° of flexion with a residual extension lag of 5°, a finding that may be observed during early rehabilitation following extensor mechanism repair. Clinical photographs demonstrated satisfactory knee flexion (Figure 8) and extension (Figure 9). At this stage, the hinged knee brace was discontinued, and progressive strengthening was initiated.

## Discussion

Patellar tendon (PT) ruptures are rare but functionally significant injuries that result in loss of active knee extension and substantial impairment [1-2]. Early surgical repair is strongly recommended, as delayed management is associated with inferior outcomes, tendon retraction, and the potential need for more complex reconstruction techniques [3,6]. Acute primary repair, particularly when augmented with suture anchors, cerclage wires, synthetic tapes, autografts, or *Internal*Brace™ constructs, demonstrates superior biomechanical stability, decreased gap formation, and low rates of re-rupture [4-5,7-8]. Similarly, Larson and Simonian highlighted the value of biological reinforcement using semitendinosus tendon augmentation, allowing immediate postoperative mobilization while maintaining repair integrity [9]. This principle parallels modern augmentation strategies using synthetic tapes or allografts to balance stability and early functional recovery.

Biomechanical studies support the use of suture anchors and *Internal*Brace™ augmentation to optimize construct strength, including improved resistance to gap formation under cyclic loading, thereby facilitating early rehabilitation [4-5,8]. Suture anchor repair reduces re-rupture risk compared with transosseous techniques, while augmentation with polyethylene tapes or cerclage wires enhances cyclic loading resistance and preserves tendon apposition at the patellar footprint [4,7]. Early mobilization, guided by cyclic loading tolerances, is crucial for collagen remodeling and functional recovery.

Chronic or neglected PT ruptures present additional challenges due to tendon retraction, poor tissue quality, and scarring, necessitating autograft or allograft augmentation to restore functional extensor mechanism integrity [3,6]. Outcomes in chronic reconstructions are generally inferior to acute repairs, with a higher likelihood of residual extensor lag and limited return to high-impact activities. Nevertheless, careful graft selection and augmentation strategies can achieve satisfactory functional recovery. Akpinar et al. reported that although knee extensor mechanism reconstruction (KEMR) provides excellent restoration of clinical function, long-term biomechanical assessments demonstrate residual weakness in peak torque and work generation compared with native tissue. These findings underscore the importance of early repair when feasible, as primary repair with biological or synthetic augmentation preserves native tendon biomechanics more effectively than full reconstruction [11].

Our case exemplifies the application of these principles in a 56-year-old male with a midsubstance patellar tendon rupture following a ground-level fall. Imaging confirmed complete rupture with proximal patellar displacement, poor tissue quality, and associated retinacular and muscular injuries. The patient underwent end-to-end suture anchor repair, circumferential Achilles tendon allograft augmentation, and *Internal*Brace™ stabilization, reflecting a multimodal approach to optimize mechanical stability and facilitate early rehabilitation. The intraoperative assessment emphasized careful evaluation of tissue quality, restoration of patellar height, and avoidance of overconstraint at the patellofemoral joint. This approach aligns with current evidence supporting augmented repairs in cases with midsubstance tears or compromised tissue [2,4,8].

Postoperative rehabilitation strategies focus on early protected range of motion, progressive weight bearing, and quadriceps strengthening, allowing high rates of return to function while minimizing complications such as stiffness or re-rupture [1,10]. Although functional outcomes are generally favorable, return to pre-injury levels of sport may be limited, particularly in older patients or those with comorbidities [6,10].

Overall, acute PT repair, particularly when guided by biomechanical principles and augmented appropriately, results in reliable restoration of knee extension, functional improvement, and the potential for early rehabilitation. The presented case demonstrates the practical integration of suture anchors, *Internal*Brace™ constructs, and allograft augmentation to manage complex midsubstance ruptures effectively. Future studies should focus on long-term outcomes, comparative effectiveness of augmentation strategies, and standardized protocols for midsubstance and chronic PT injuries.

## Conclusions

Acute midsubstance patellar tendon ruptures can be effectively managed with primary repair augmented by suture anchors, *Internal*Brace™ constructs, and, when necessary, allograft reinforcement. This combined approach restores tendon continuity, maintains patellar height, and provides biomechanical stability sufficient to allow early mobilization. Clinical outcomes are favorable, with low rates of re-rupture and extensor lag, even in patients with poor tissue quality. Augmentation strategies, including *Internal*Brace™ and allograft reinforcement, offer valuable options for challenging midsubstance tears, supporting early rehabilitation and optimizing functional recovery. Further studies with larger cohorts are needed to confirm long-term outcomes and the comparative benefits of different augmentation techniques.
